# Remote ischemic preconditioning and clinical outcomes after pediatric cardiac surgery: a systematic review and meta-analysis

**DOI:** 10.1186/s12871-023-02064-6

**Published:** 2023-04-01

**Authors:** Jianwen Li, Xiwen Wang, Wengui Liu, Shihong Wen, Xueping Li

**Affiliations:** 1 Departments of Anesthesiology, DongGuan SongShan Lake Tungwah Hospital, DongGuan, China; 2 Departments of Anesthesiology, The First Affiliated Hospital, Sun Yat-Sen University, Guangzhou, China

**Keywords:** Cardiac surgery, Children, remote ischemic preconditioning, Propofol

## Abstract

**Background:**

The benefit of remote ischemia preconditioning (RIPreC) in pediatric cardiac surgery is unclear. The objective of this systematic review and meta-analysis was to examine the effectiveness of RIPreC in reducing the duration of mechanical ventilation and intensive care unit (ICU) length of stay after pediatric cardiac surgery.

**Methods:**

We searched PubMed, EMBASE and the Cochrane Library from inception to December 31, 2022. Randomized controlled trials comparing RIPreC versus control in children undergoing cardiac surgery were included. The risk of bias of included studies was assessed using the Risk of Bias 2 (RoB 2) tool. The outcomes of interest were postoperative duration of mechanical ventilation and ICU length of stay. We conducted random-effects meta-analysis to calculate weighted mean difference (WMD) with 95% confidence interval (CI) for the outcomes of interest. We performed sensitivity analysis to examine the influence of intraoperative propofol use.

**Results:**

Thirteen trials enrolling 1,352 children were included. Meta-analyses of all trials showed that RIPreC did not reduce postoperative duration of mechanical ventilation (WMD -5.35 h, 95% CI -12.12–1.42) but reduced postoperative ICU length of stay (WMD -11.48 h, 95% CI -20.96– -2.01). When only trials using propofol-free anesthesia were included, both mechanical ventilation duration (WMD -2.16 h, 95% CI -3.87– -0.45) and ICU length of stay (WMD -7.41 h, 95% CI -14.77– -0.05) were reduced by RIPreC. The overall quality of evidence was moderate to low.

**Conclusions:**

The effects of RIPreC on clinical outcomes after pediatric cardiac surgery were inconsistent, but both postoperative mechanical ventilation duration and ICU length of stay were reduced in the subgroup of children not exposed to propofol. These results suggested a possible interaction effect of propofol. More studies with adequate sample size and without intraoperative propofol use are needed to define the role of RIPreC in pediatric cardiac surgery.

**Supplementary Information:**

The online version contains supplementary material available at 10.1186/s12871-023-02064-6.

## Background

Remote ischemic preconditioning (RIPreC) has long been viewed as an attractive approach to mitigate the ischemia–reperfusion injury to heart and other organs induced by cardiopulmonary bypass. Despite some beneficial effects in terms of reductions in biomarkers of organ injury [1], most randomized controlled trials (RCT) failed to show a benefit of RIPreC on clinical outcomes in patients undergoing cardiac surgery [2, 3]. The most frequently discussed confounding factor is the concomitant use of intravenous anesthetic propofol [4], which could interfere and inhibit RIPreC’s protective effects [5, 6]. In addition, advanced age and comorbidities such as diabetes and hypertension were also reported to influence RIPreC-induced organ protection [7, 8]. Taking all these into consideration, we hypothesized that RIPreC may bring benefit on clinical outcomes in children receiving cardiac surgery under propofol-free anesthesia.

Previous meta-analyses did not show significant cardioprotective effect of RIPreC in pediatric cardiac surgery [9, 10]. However, the included RCTs had small sample sizes, and the confounding effect of concomitant propofol use was not explored. Recently, several new trials have been published; the information size regarding this issue has increased markedly. We therefore conducted an updated meta-analysis focusing on the effects of RIPreC on clinical outcomes in relation to intraoperative propofol use. The objective of this systematic review and meta-analysis was to examine the effectiveness of RIPreC in reducing the duration of mechanical ventilation and intensive care unit (ICU) length of stay after pediatric cardiac surgery, and to explore the impact of propofol on the effectiveness of RIPreC.

## Methods

### Literature search and study selection

This study was conducted and reported according to the Cochrane handbook and the PRISMA statement [11, 12]. The PRISMA 2020 checklist is provided in Supplementary Table 1. The study protocol was developed a priori and was not changed during the study, but was not registered. We searched PubMed, EMBASE via Ovid and the Cochrane Library via Ovid from inception to December 31, 2022 using free-text representing RIPreC, cardiac surgery and children. The keywords used in our search of the PubMed database were (cardiac OR heart) AND (surgery OR operation OR preoperative OR intraoperative OR perioperative) AND (preconditioning) AND (child^*^ OR paediatric OR pediatric OR infant^*^ OR young OR neonate^*^) AND (RCT OR randomized controlled trial OR Random^*^). The full search strategies for all databases are provided in detail (Supplementary Table 2). RCTs that met the following criteria were included: [1] population: children (< 18 yrs) undergoing any cardiac surgery; [2] intervention: standard care plus RIPreC vs standard care with/without a sham procedure; [3] outcome: postoperative mechanical ventilation duration, intensive care unit (ICU) length of stay, or both, was reported. Two reviewers (JL, XW) screened each record and each report retrieved, whether they worked independently. A third reviewer (SW) was consulted when there was disagreement.

### Data extraction and quality assessment

Two reviewers (JL, XW) collected data from each report independently. Main characteristics of trial design, patients, interventions, and outcomes in each eligible RCT were recorded. Important missing or unclear data were obtained or confirmed by contacting the study investigators using emails. The outcomes of interest of this study were postoperative mechanical ventilation duration and ICU length of stay (expressed in hours). They were chosen as measures of effectiveness because prolonged mechanical ventilation and ICU stay are usually consequences of cardiopulmonary dysfunction or slower recovery after cardiac surgery and because they are powerful predictors of both short- and long-term mortality [13, 14]. All outcome data expressed in other units were converted to hours. The methodological quality of included RCTs was assessed using the Risk of Bias 2 (RoB 2) tool. [12] by two independent reviewers.

### Statistical analysis

Using Review Manager 5 (Cochrane Collaboration, Copenhagen, Denmark), we conducted random-effects meta-analysis to calculate weighted mean difference (WMD) with 95% confidence interval (CI) for the outcomes of interest. Where data were reported as medians, they were converted to means and standard deviations [15]. Heterogeneity was assessed using chi-square test, and was quantified by I^2^ statistic. Publication bias was visually assessed by inspecting funnel plots and statistically tested using Egger's test. TSA was conducted using a specific software (User Manual for TSA; Copenhagen Trial Unit 2011, Copenhagen, Denmark) [16]. Our assumptions included two-sided testing, type I error of 5%, and power of 80%. A priori planned sensitivity analysis was conducted by including only RCTs with no intraoperative propofol use. Unless an anesthetic regimen without propofol was detailed, it was assumed that propofol was administered. We used GRADE (Grading of Recommendations Assessment, Development and Evaluation) system for evaluating the overall quality of evidence. The quality of evidence is assessed based on factors including the study design, the risk of bias, consistency, directness and precision.

## Results

### Literature search findings

**Fig. 1:**
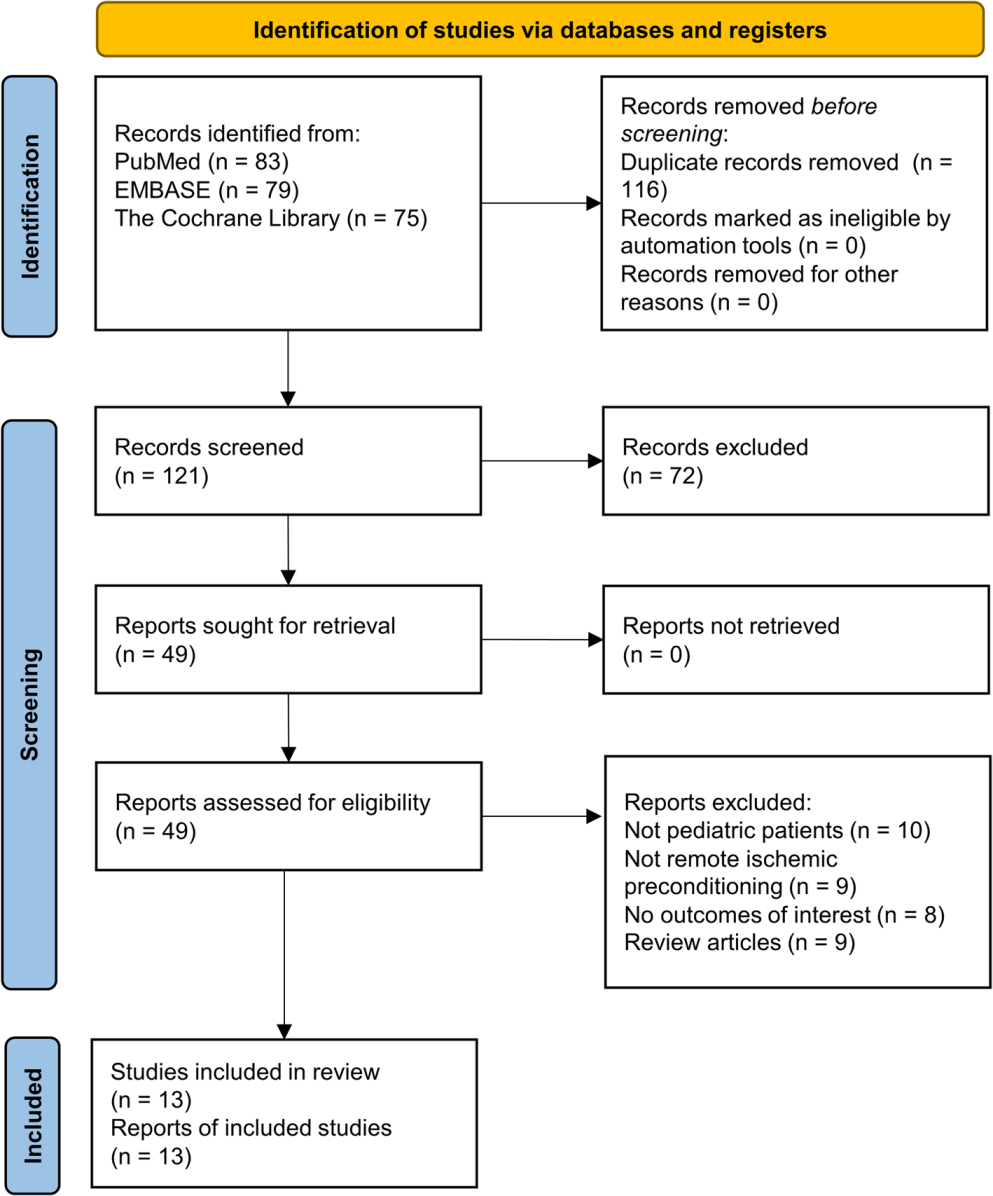
PRISMA flow diagram of the study

**Fig. 2:**
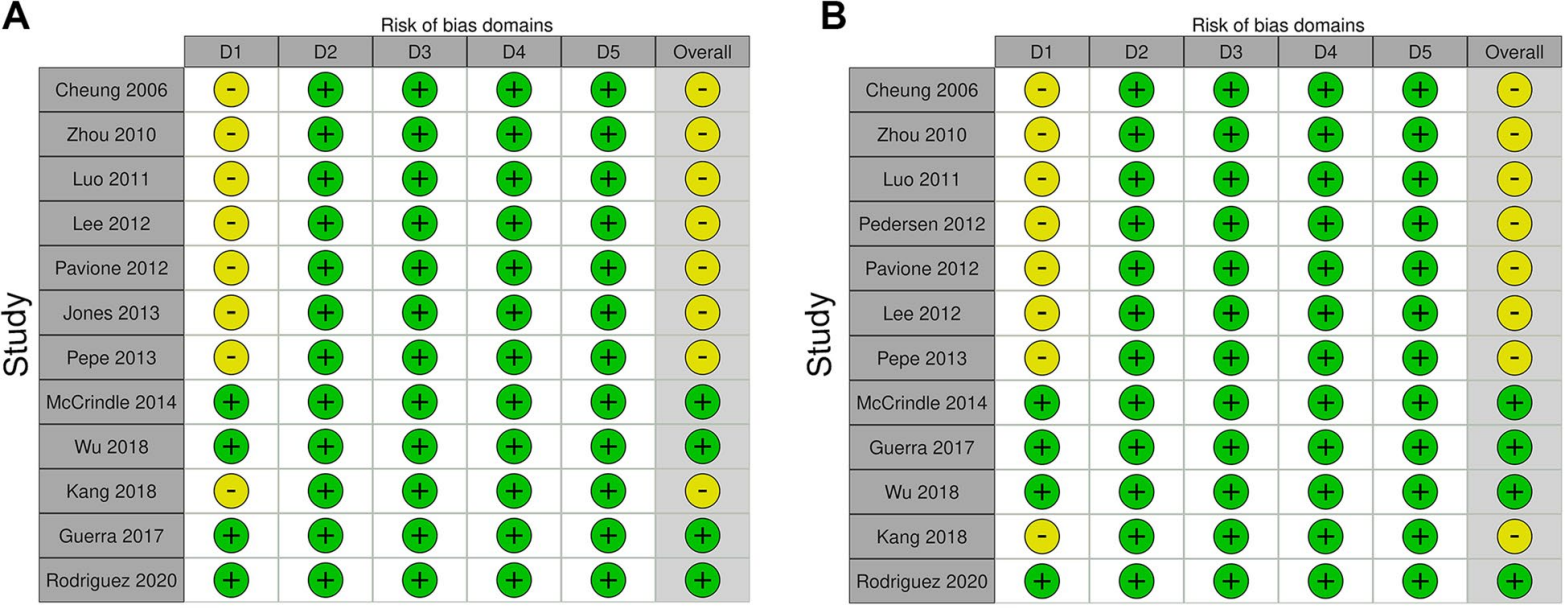
Risk of bias assessment of included RCTs on the outcomes duration of mechanical ventilation (**A**) and length of ICU stay (**B**). Domains: D1: Bias arising from the randomization process. D2: Bias due to deviations from intended intervention. D3: Bias due to missing outcome data. D4: Bias in measurement of the outcome. D5: Bias in selection of the reported result. Judgement: -: Some concerns, +: Low.

Figure 1 is a flow diagram of the study and summarizes the process of trial selection. Thirteen eligible RCTs were identified that had enrolled 1,352 children randomly assigned to either RIPreC or control groups [17–29]. The main characteristics and methodological quality of these trials are summarized in Table 1 and Fig. 2. The studies that were excluded from this review and the reasons were provided in Supplementary Table 3.

**Table 1:** Main characteristics of included trials

**Study**	**Country**	**No** of **pts**	**Age (RIPreC / control)**	**Type of heart disease**	**Timing of RIPreC**	**RIPreC site, duration (cycles × min) and pressure**	**Propofol use**	**Risk of bias**
Cheung 2006 [17]	Canada	37	0.9 / 2.2 years	CHD	5–10 min before CPB	LL, 4 × 5, 15 mmHg > SAP	No	Unclear
Zhou 2010 [18]	China	60	5.4 / 5.1 months	VSD	24 h and 1 h before surgery	UL, 3 × 5, 240 mmHg	Unknown	Unclear
Luo 2011[19]	China	40	2.2 / 3.1 years	VSD	Immediately after anesthesia induction	LL, 3 × 5, 200 mmHg	No	Unclear
Lee 2012 [20]	Korea	55	3.7 / 3.4 months	VSD	~ 10 min after anesthesia induction	LL, 4 × 5, 30 mmHg > SAP	No	Unclear
Pavione 2012 [21]	Brazil	22	5.8 / 6.1 months	CHD	24 h before surgery	LL, 4 × 5, 15 mmHg > SAP	No	Unclear
Pedersen 2012 [22]	Denmark	105	1.0 / 0.9 years	CHD	Immediately after anesthesia induction	LL, 4 × 5, 40 mmHg > SAP	Yes	High
Jones 2013 [23]	Australia	39	8.1 / 5.5 days	TGA, HLHS	After anesthesia induction	LL, 4 × 5, 15 mmHg > SAP	No	Unclear
Pepe 2013 [24]	Australia	40	7.6 / 7.4 months	ToF	Immediately after anesthesia induction	LL, 4 × 5, 30 mmHg > SAP	No	Unclear
McCrindle 2014 [25]	Canada	299	2.2 / 3.1 years	CHD	During anesthesia induction	LL, 4 × 5, 15 mmHg > SAP	Yes	Unclear
Guerra 2017 [26]	Canada	45	7.5 / 13.7 days	CHD	24–48 h before surgery and intra-operatively before CPB	LL, 4 × 5, 20 mmHg > SAP	No	Low
Wu 2018 [27]	China	112	10.5 / 11.2 months	ToF	After anesthesia, 55–65 min before CPB	LL, 3 × 5, 30 mmHg > SAP	No	Low
Kang 2018 [28]	China	449	3.3 / 2.6 years	CHD	12 h before surgery	LL, 4 × 5, 30 mmHg > SAP	Unknown	High
Rodriguez 2020 [29]	UK	49	19 / 9 months	CHD	15–20 h before surgery and after anaesthesia induction prior to surgery	UL or LL, 3 × 5, 20 mmHg > SAP	Unknown	Low

### Duration of mechanical ventilation

**Fig. 3:**
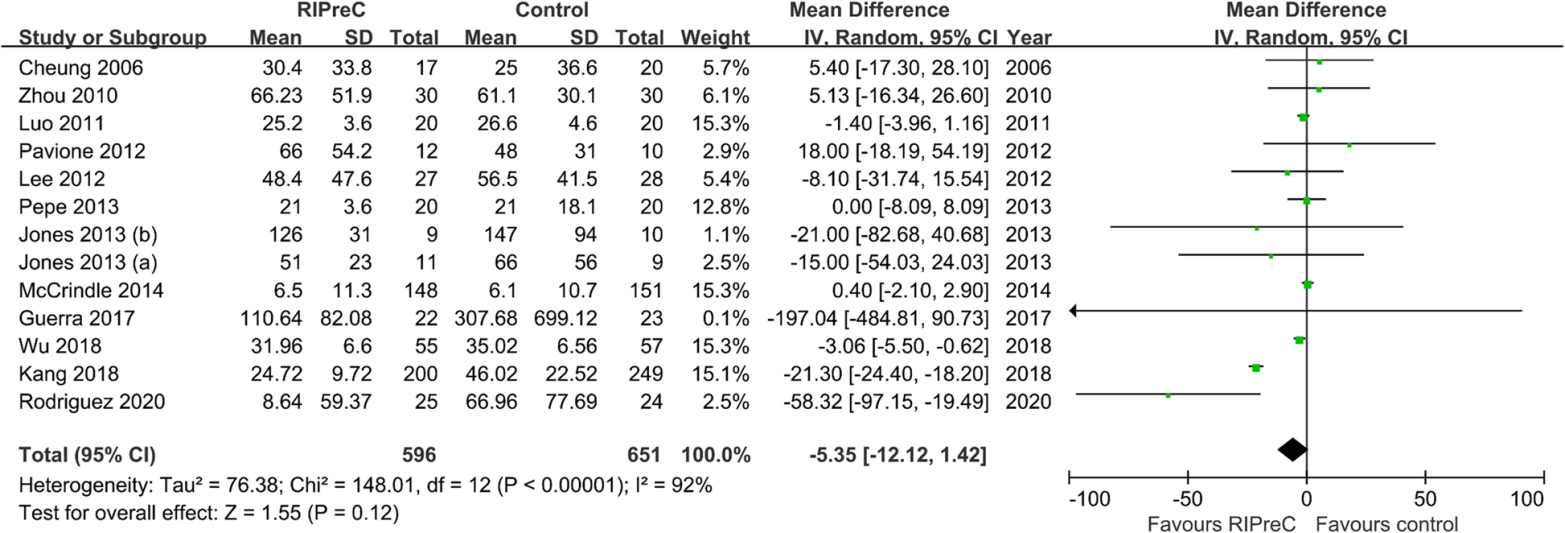
Forest plot of the effect of RIPreC on postoperative duration of mechanical ventilation (overall cohort)

**Fig. 4:**
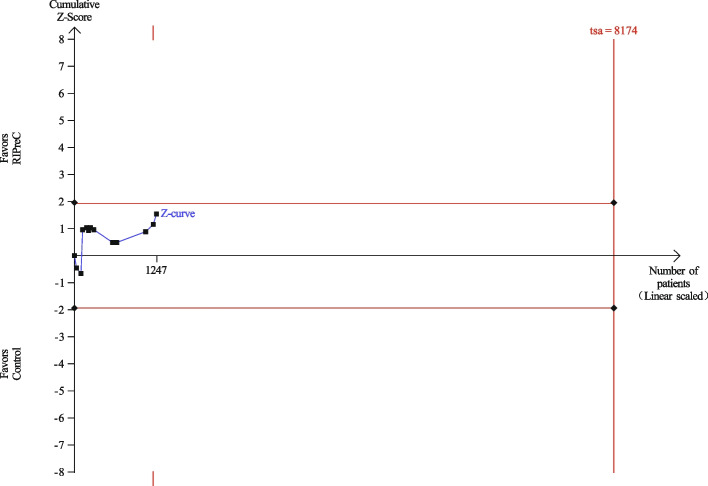
Trial sequential analysis plot of postoperative duration of mechanical ventilation (overall cohort). Trial sequential analysis of 11 RCTs that compared RIPreC versus control on postoperative duration of mechanical ventilation. The cumulative z curve did not cross the conventional boundary. The information size was too small to produce the inner wedge futility area, indicating the current evidence is inconclusive. A required information size of 8,174 patients was calculated using α = 0.05 (two sided), β = 0.20 (power 80%) and the mean difference generated in the conventional meta-analysis

**Fig. 5:**
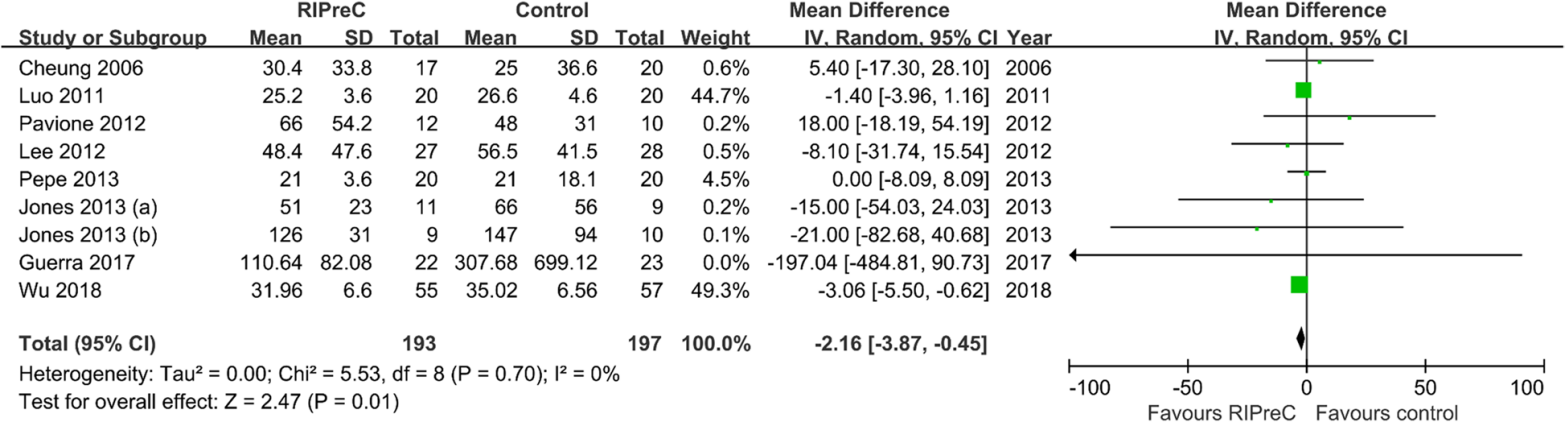
Forest plot of the effect of RIPreC on postoperative duration of mechanical ventilation (sensitivity analysis)

**Fig. 6:**
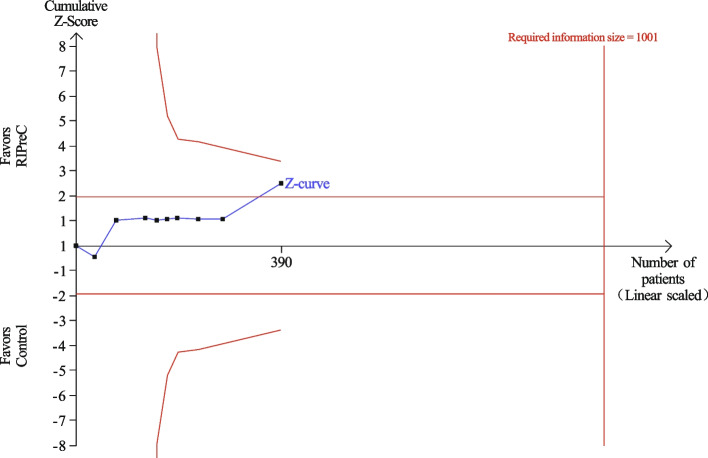
Trial sequential analysis plot of postoperative duration of mechanical ventilation (sensitivity analysis). Trial sequential analysis of 8 RCTs that did not use propofol anesthesia on postoperative duration of mechanical ventilation. The cumulative z curve crossed the conventional boundary but not the trial sequential monitoring boundary for benefit, indicating the current evidence is inconclusive. A required information size of 1,001 patients was calculated using α = 0.05 (two sided), β = 0.20 (power 80%) and the mean difference generated in the conventional meta-analysis

Twelve RCTs reported postoperative mechanical ventilation duration in a total of 1,247 children. Overall, RIPreC did not reduced the duration of mechanical duration (WMD -5.35 h, 95% CI -12.12–1.42; I^2^ = 92%; Fig. 3). TSA suggested the current evidence was inconclusive and the required information size to draw a firm conclusion would be 8,174 (Fig. 4). In the sensitivity analysis including only RCTs with no intraoperative propofol use, RIPreC significantly reduced the duration of postoperative mechanical duration (WMD -2.16 h, 95% CI -3.87– -0.45; I^2^ = 0%; Fig. 5). Meanwhile a marked reduction in study heterogeneity was observed. TSA again suggested the result was inconclusive, but the required information size reduced to 1,001 (Fig. 6).

### ICU length of stay

**Fig. 7:**
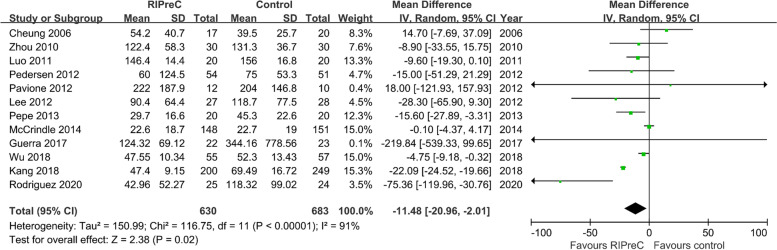
Forest plot of the effect of RIPreC on postoperative ICU length of stay (overall cohort)

**Fig. 8:**
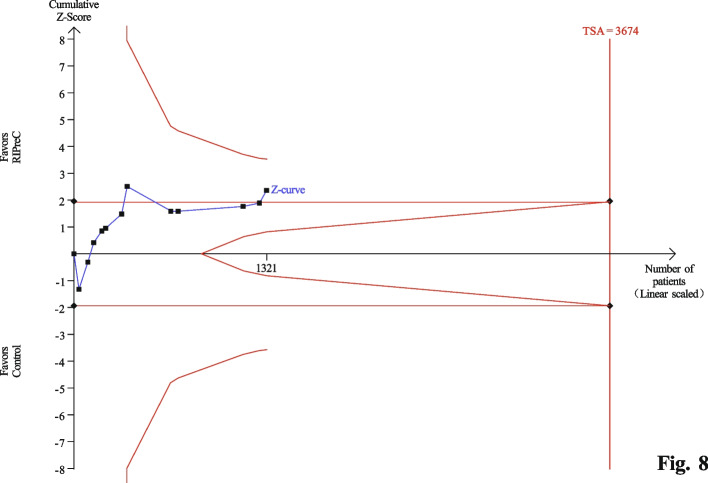
Trial sequential analysis plot of postoperative ICU length of stay (overall cohort). Trial sequential analysis of 11 RCTs that compared RIPreC versus control on postoperative ICU length of stay. The cumulative z curve crossed the conventional boundary but not the trial sequential monitoring boundary for benefit, indicating the current evidence is inconclusive. A required information size of 3,674 patients was calculated using α = 0.05 (two sided), β = 0.20 (power 80%) and the mean difference generated in the conventional meta-analysis

**Fig. 9:**
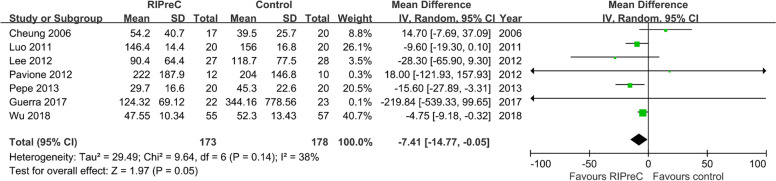
Forest plot of the effect of RIPreC on postoperative ICU length of stay (sensitivity analysis)

**Fig. 10:**
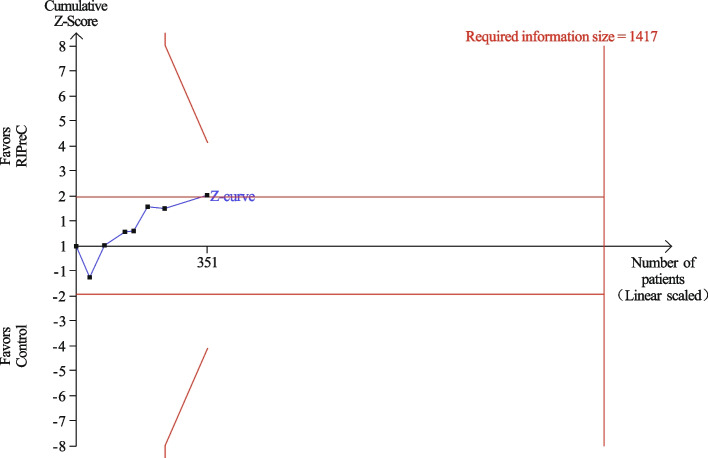
Trial sequential analysis plot of postoperative ICU length of stay (sensitivity analysis). Trial sequential analysis of 7 RCTs that did not use propofol anesthesia on postoperative ICU length of stay. The cumulative z curve crossed the conventional boundary but not the trial sequential monitoring boundary for benefit, indicating the current evidence is inconclusive. A required information size of 1,417 patients was calculated using α = 0.05 (two sided), β = 0.20 (power 80%) and the mean difference generated in the conventional meta-analysis

Twelve RCTs enrolling a total of 1,313 children reported postoperative ICU length of stay. Overall, RIPreC reduced ICU length of stay (WMD -11.48 h, 95% CI -20.96 – -2.01; I^2^ = 91%; Fig. 7). TSA suggested the current evidence was inconclusive and the required information size to draw a firm conclusion would be 3,674 (Fig. 8). In the sensitivity analysis including only RCTs with no intraoperative propofol use, RIPreC significantly reduced postoperative ICU length of stay (WMD -7.41 h, 95% CI -14.77– -0.05; I^2^ = 38%; Fig. 9). A markedly reduced heterogeneity was also seen. TSA again suggested the result was inconclusive, but the required information size reduced to 1,417, Fig. 10).

### Publication bias and GRADE evidence profile

**Fig. 11:**
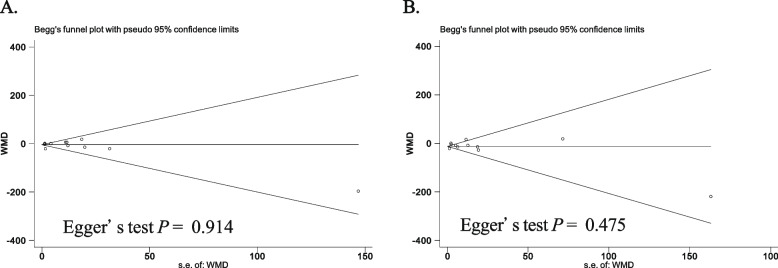
Funnel plots for assessment of publication bias in mechanical ventilation duration (**A**) and ICU length of stay (**B**)

No evidence of publication bias was detected (Fig. 11). The GRADE evidence profile for the outcomes is shown in Table 2.

**Table 2:** GRADE evidence profile

**Quality assessment**												**No of patients**					**Effect**			**Quality**		**Importance**
**No of studies**	**Design**		**Risk of bias**			**Inconsistency**		**Indirectness**			**Imprecision**	**RIPreC**			**Control**		**Absolute (mean difference)**					
**Mechanical ventilation duration (overall cohort)**																						
12	randomised trials		serious1			serious2		no serious indirectness			no serious imprecision	596			654		5.35 h shorter (12.12 h shorter to 1.42 h longer)			⊕ ⊕ ◯⃝ LOW		IMPORTANT
**Mechanical ventilation duration (in the absence of propofol)**																						
8	randomised trials		serious3			no serious inconsistency		no serious indirectness			no serious imprecision	193			197		2.16 h shorter (3.87 h to 0.45 h shorter)			⊕ ⊕ ⊕ ◯MODERATE		IMPORTANT
**ICU length of stay (overall cohort)**																						
12	randomised trials		serious1			serious4		no serious indirectness			no serious imprecision	630			683		11.48 h shorter (20.96 h shorter to 2.01 h shorter)			⊕ ⊕ ◯⃝LOW		IMPORTANT
**ICU length of stay (in the absence of propofol)**																						
7	randomised trials		serious3			no serious inconsistency		no serious indirectness			no serious imprecision	173			178		7.41 h shorter (14.77 h to 0.05 h shorter)			⊕ ⊕ ◯⃝ MODERATE		IMPORTANT

*AbbreviationsCHD* Congenital heart disease, *CPB* Cardiopulmonary bypass *HLHS* Hypoplastic left heart syndrome, *LL* Low limb, *RIPreC* Remote ischemic preconditioning, *SAP* Systolic arterial pressure, *TGA* Transposition of the great arteries, *ToF* Tetralogy of Fallot, *UL* Upper limb, *VSD* Ventricular septal defect

## Discussion

In this meta-analysis of 13 RCTs and 1,352 children, the effects of RIPreC on decreasing postoperative mechanical ventilation duration and ICU length of stay were inconsistent in the overall cohort. However, we found significant improvement in these outcomes when only trials using propofol-free anesthesia were analyzed, and the heterogeneity among studies were substantially reduced. Although a firm conclusion could not be reached due to inadequate information size, our findings support the idea that propofol may interfere with the protective effects of RIPreC.

The mechanism of RIPreC-induced organ protection is complex and involves both humoral and sensory-neuronal pathways [30]. Several theories explaining how propofol might influence these pathways have been proposed. Propofol has been reported to abrogates myocardial STAT 5 phosphorylation, impair sensory fiber activation, and interfere with central nervous control of cardiac vagal nerves [31]. All these are important for the cardioprotection by RIPreC. Another theory is that the anti-inflammatory and antioxidant properties of propofol could obscure the effects of RIPreC. In congruent with these theories, RIPreC has been shown to reduce morbidity and mortality after adult cardiac surgery when combined with volatile anesthesia but not total intravenous (propofol) anesthesia in meta-analyses [32, 33].

The current study had several limitations. First, most included trials had a small sample size, a short follow-up duration, and were only powered to detect differences in surrogate endpoints such as blood biomarkers. Postoperative mortality and major complications had low incidence and were inconsistently reported. We could not evaluate the effects of RIPreC on these important outcomes. Second, substantial heterogeneity was noted across the included trials with regard to the age of children, the type and severity of heart disease, and the protocol of RIPreC. These factors contributed to the high variance in outcome data distribution. We therefore used random-effects model for this meta-analysis. We also performed a post hoc analysis by using standardized mean difference as the effect measure, and the result was consistent with the primary analysis. Third, we were unable to perform statistical test for the possible interaction effect of propofol because the number of trials were limited and because the proportions of propofol use in some trials were unknown. Thus, the effect of propofol could not be confirmed. Fourth, the protocol of this systematic review and meta-analysis was not registered a priori, and it is best to use a validated search filter For RCTs search.

Despite these limitations, our study provides additional evidence that RIPreC may show clinically significant effects in cardiac surgery when propofol anesthesia is not used. The findings of this study provide insights for the design of future researches. Above all, considering the possible confounding effect of propofol and the realizability of required sample size calculated by TSA, it is mandatory for future trials to avoid propofol as part of the anesthesia regimen. In addition, future trials should be adequately powered for clinically important outcomes such as ICU length of stay, rather than merely surrogate outcomes. Since the incidence of postoperative short-term mortality and major complications are low, longer follow-up durations are needed to evaluate the long-term effects of RIPreC on pediatric cardiac surgery.

## Conclusions

RIPreC does not reduce postoperative mechanical ventilation duration or ICU length of stay after pediatric cardiac surgery. In trials that did not use propofol, significant reductions in mechanical ventilation duration and ICU length of stay were observed, suggesting that propofol may interfere with the protective effects of RIPreC. Future trials with adequate power are needed to evaluate the independent role of RIPreC in pediatric cardiac surgery under propofol-free anesthesia.

## Supplementary Information

**Additional file 1.**

## Data Availability

All data generated or analyzed during this study are included in this published article.

## Abbreviations

RIPreC: Remote ischemia preconditioning
ICU: Intensive care unit
TSA: Trial sequential analysis
WMD: Weighted mean difference
