# Supplementary material for: Remote ischemic preconditioning and clinical outcomes after pediatric cardiac surgery: a systematic review and meta-analysis
Source: BMC Anesthesiol. 2023 Apr 1;23:105. doi: 10.1186/s12871-023-02064-6 (PMC10067320; doi:10.1186/s12871-023-02064-6)
Supplement: Supplementary file 1 — Additional file 1. [file 12871_2023_2064_MOESM1_ESM.pdf]

Supplementary Table 1. The PRISMA 2020 checklist

Section and Topic	Item #	Checklist item	Location where item is reported
**TITLE**			
Title	1	Identify the report as a systematic review.	page 1
**ABSTRACT**			
Abstract	2	See the PRISMA 2020 for Abstracts checklist.	page 2-3
**INTRODUCTION**			
Rationale	3	Describe the rationale for the review in the context of existing knowledge.	page 4
Objectives	4	Provide an explicit statement of the objective(s) or question(s) the review addresses.	page 4
**METHODS**			
Eligibility criteria	5	Specify the inclusion and exclusion criteria for the review and how studies were grouped for the syntheses.	page 5
Information sources	6	Specify all databases, registers, websites, organisations, reference lists and other sources searched or consulted to identify studies. Specify the date when each source was last searched or consulted.	page 5
Search strategy	7	Present the full search strategies for all databases, registers and websites, including any filters and limits used.	Suppl Table 2
Selection process	8	Specify the methods used to decide whether a study met the inclusion criteria of the review, including how many reviewers screened each record and each report retrieved, whether they worked independently, and if applicable, details of automation tools used in the process.	page 5
Data collection process	9	Specify the methods used to collect data from reports, including how many reviewers collected data from each report, whether they worked independently, any processes for obtaining or confirming data from study investigators, and if applicable, details of automation tools used in the	page 5

Section and Topic	Item #	Checklist item	Location where item is reported
		process.	
Data items	10a	List and define all outcomes for which data were sought. Specify whether all results that were compatible with each outcome domain in each study were sought (e.g. for all measures, time points, analyses), and if not, the methods used to decide which results to collect.	page 5
	10b	List and define all other variables for which data were sought (e.g. participant and intervention characteristics, funding sources). Describe any assumptions made about any missing or unclear information.	page 5
Study risk of bias assessment	11	Specify the methods used to assess risk of bias in the included studies, including details of the tool(s) used, how many reviewers assessed each study and whether they worked independently, and if applicable, details of automation tools used in the process.	page 5
Effect measures	12	Specify for each outcome the effect measure(s) (e.g. risk ratio, mean difference) used in the synthesis or presentation of results.	page 6
Synthesis methods	13a	Describe the processes used to decide which studies were eligible for each synthesis (e.g. tabulating the study intervention characteristics and comparing against the planned groups for each synthesis (item #5)).	page 6
	13b	Describe any methods required to prepare the data for presentation or synthesis, such as handling of missing summary statistics, or data conversions.	page 6
	13c	Describe any methods used to tabulate or visually display results of individual studies and syntheses.	page 6
	13d	Describe any methods used to synthesize results and provide a rationale for the choice(s). If meta-analysis was performed, describe the model(s), method(s) to identify the presence and extent of statistical heterogeneity, and software package(s) used.	page 6
	13e	Describe any methods used to explore possible causes of heterogeneity among study results (e.g. subgroup analysis, meta-regression).	page 6
	13f	Describe any sensitivity analyses conducted to assess robustness of the synthesized results.	page 6
Reporting bias assessment	14	Describe any methods used to assess risk of bias due to missing results in a synthesis (arising from reporting biases).	page 6

Section and Topic	Item #	Checklist item	Location where item is reported
Certainty assessment	15	Describe any methods used to assess certainty (or confidence) in the body of evidence for an outcome.	page 6
**RESULTS**			
Study selection	16a	Describe the results of the search and selection process, from the number of records identified in the search to the number of studies included in the review, ideally using a flow diagram.	page 7, fig 1
	16b	Cite studies that might appear to meet the inclusion criteria, but which were excluded, and explain why they were excluded.	Suppl Table 2
Study characteristics	17	Cite each included study and present its characteristics.	table 1
Risk of bias in studies	18	Present assessments of risk of bias for each included study.	fig 2
Results of individual studies	19	For all outcomes, present, for each study: (a) summary statistics for each group (where appropriate) and (b) an effect estimate and its precision (e.g. confidence/credible interval), ideally using structured tables or plots.	page 7-8
Results of syntheses	20a	For each synthesis, briefly summarise the characteristics and risk of bias among contributing studies.	page 7-8
	20b	Present results of all statistical syntheses conducted. If meta-analysis was done, present for each the summary estimate and its precision (e.g. confidence/credible interval) and measures of statistical heterogeneity. If comparing groups, describe the direction of the effect.	page 7-8
	20c	Present results of all investigations of possible causes of heterogeneity among study results.	page 7-8
	20d	Present results of all sensitivity analyses conducted to assess the robustness of the synthesized results.	page 7-8
Reporting biases	21	Present assessments of risk of bias due to missing results (arising from reporting biases) for each synthesis assessed.	page 7-8

Section and Topic	Item #	Checklist item	Location where item is reported
Certainty of evidence	22	Present assessments of certainty (or confidence) in the body of evidence for each outcome assessed.	page 8
**DISCUSSION**			
Discussion	23a	Provide a general interpretation of the results in the context of other evidence.	page 9
	23b	Discuss any limitations of the evidence included in the review.	page 9-10
	23c	Discuss any limitations of the review processes used.	page 9-10
	23d	Discuss implications of the results for practice, policy, and future research.	page 10
**OTHER INFORMATION**			
Registration and protocol	24a	Provide registration information for the review, including register name and registration number, or state that the review was not registered.	page 5
	24b	Indicate where the review protocol can be accessed, or state that a protocol was not prepared.	page 5
	24c	Describe and explain any amendments to information provided at registration or in the protocol.	page 5
Support	25	Describe sources of financial or non-financial support for the review, and the role of the funders or sponsors in the review.	page 12
Competing interests	26	Declare any competing interests of review authors.	page 12
Availability of data, code and other materials	27	Report which of the following are publicly available and where they can be found: template data collection forms; data extracted from included studies; data used for all analyses; analytic code; any other materials used in the review.	page 12

Supplementary Table 2. Database search strategy

PubMed (n=83 on December 31, 2022)

(cardiac OR heart) AND (surgery OR operation OR preoperative OR intraoperative OR perioperative) AND (preconditioning) AND (child* OR paediatric OR pediatric OR infant* OR young OR neonate*) AND (RCT OR randomized controlled trial OR Random*) in all fields

EMBASE (n=79 on December 31, 2022)

('cardiac' OR 'heart'/exp) AND ('surgery'/exp OR 'operation'/exp OR 'preoperative' OR 'intraoperative' OR 'perioperative') AND ('preconditioning') AND ('child*' OR 'paediatric' OR 'pediatric' OR 'infant*' OR 'young' OR 'neonate*') AND ('rct' OR 'randomized controlled trial'/exp OR 'random*') in all fields

Cochrane Library (n=75 on December 31, 2022)

(cardiac OR heart) AND (surgery OR operation OR preoperative OR intraoperative OR perioperative) AND (preconditioning) AND (child* OR paediatric OR pediatric OR infant* OR young OR neonate*) AND (RCT OR randomized controlled trial OR Random*) in Title Abstract Keywords in Trials

Supplementary Table 3. Excluded studies with reasons

Not pediatric patients

- Li G, Chen S, Lou W, Jiang H. The protective effects of cardiac ischemic preconditioning on lung in cardiac operation with cardiopulmonary bypass. Hunan Yi Ke Da Xue Xue Bao. 1998;23(1):41-3
- Venugopal V, Hausenloy DJ, Ludman A, Di Salvo C, Kolvekar S, Yap J, Lawrence D, Bognolo J, Yellon DM. Remote ischaemic preconditioning reduces myocardial injury in patients undergoing cardiac surgery with cold-blood cardioplegia: a randomised controlled trial. Heart. 2009 Oct;95(19):1567-71
- Williams JM, Young P, Pilcher J, Weatherall M, Miller JH, Beasley R, La Flamme AC. Remote ischaemic preconditioning does not alter perioperative cytokine production in high-risk cardiac surgery. Heart Asia 2012 Aug 13;4(1):97-101
- Young PJ, Dalley P, Garden A, Horrocks C, La Flamme A, Mahon B, Miller J, Pilcher J, Weatherall M, Williams J, Young W, Beasley R. A pilot study investigating the effects of remote ischemic preconditioning in high-risk cardiac surgery using a randomised controlled double-blind protocol. Basic Res Cardiol. 2012 May;107(3):256
- Luca MC, Liuni A, McLaughlin K, Gori T, Parker JD. Daily ischemic preconditioning provides sustained protection from ischemia-reperfusion induced endothelial dysfunction: a human study. J Am Heart Assoc 2013 Feb 22;2(1):e000075.

- Cho YJ, Lee EH, Lee K, Kim TK, Hong DM, Chin JH, Choi DK, Bahk JH, Sim JY, Choi IC, Jeon Y. Long-term clinical outcomes of Remote Ischemic Preconditioning and Postconditioning Outcome (RISPO) trial in patients undergoing cardiac surgery. Int J Cardiol. 2017 Mar 15; 231:84-89.
- Struck R, Wittmann M, Müller S, Meybohm P, Müller A, Bagci S. Effect of Remote Ischemic Preconditioning on Intestinal Ischemia-Reperfusion Injury in Adults Undergoing On-Pump CABG Surgery: A Randomized Controlled Pilot Trial. J Cardiothorac Vasc Anesth. 2018 Jun;32(3):1243-1247
- Song JW, Lee WK, Lee S, Shim JK, Kim HJ, Kwak YL. Remote ischaemic conditioning for prevention of acute kidney injury after valvular heart surgery: a randomised controlled trial. Br J Anaesth. 2018 Nov;121(5):1034-1040
- Hausenloy DJ, Kharbanda R, Rahbek Schmidt M, Møller UK, Ravkilde J, Okkels Jensen L, Engstrøm T, Garcia Ruiz JM, Radovanovic N, Christensen EF, Sørensen HT, Ramlall M, Bulluck H, Evans R, Nicholas J, Knight R, Clayton T, Yellon DM, Bøtker HE. Effect of remote ischaemic conditioning on clinical outcomes in patients presenting with an ST-segment elevation myocardial infarction undergoing primary percutaneous coronary intervention. Eur Heart J 2015 Aug 1;36(29):1846-8.
- Hong DM, Lee EH, Kim HJ, Min JJ, Chin JH, Choi DK, Bahk JH, Sim JY, Choi IC, Jeon Y. Does remote ischaemic preconditioning with postconditioning improve clinical outcomes of patients undergoing cardiac surgery? Remote Ischaemic Preconditioning with Postconditioning Outcome Trial. Eur Heart J. 2014 Jan;35(3):176-83.

Not remote ischemic preconditioning

- Malagon I, Hogenbirk K, van Pelt J, Hazekamp MG, Bovill JG. Effect of three different anaesthetic agents on the postoperative production of cardiac troponin T in paediatric cardiac surgery. Br J Anaesth 2005 Jun;94(6):805-9.
- Luo W, Li B, Lin G, Chen R, Huang R. Does cardioplegia leave room for postconditioning in paediatric cardiac surgery? Cardiol Young. 2008 Jun;18(3):282-7.
- Lai VK, Ang KL, Rathbone W, Harvey NJ, Galiñanes M. Randomized controlled trial on the cardioprotective effect of bone marrow cells in patients undergoing coronary bypass graft surgery. Eur Heart J. 2009 Oct;30(19):2354-9.
- Jin Z, Duan W, Chen M, Yu S, Zhang H, Feng G, Xiong L, Yi D. The myocardial protective effects of adenosine pretreatment in children undergoing cardiac surgery: a randomized controlled clinical trial. Eur J Cardiothorac Surg 2011 May;39(5):e90-6.
- Singh P, Chauhan S, Jain G, Talwar S, Makhija N, Kiran U. Comparison of cardioprotective effects of volatile anesthetics in children undergoing ventricular septal defect closure. World J Pediatr Congenit Heart Surg. 2013 Jan;4(1):24-9.
- Mahdavi L, Abdollahi MH, Entezari A, Salehi E, Hosseini H, Moshtaghioon SH, Rafie A, Rahimianfar AA. The effect of sevoflurane versus propofol anesthesia on troponin I after congenital heart surgery, a randomized clinical trial. Adv Biomed Res. 2015 May 11;4:86

- Bettex DA, Wanner PM, Bosshart M, Balmer C, Knirsch W, Dave H, Dillier C, Bürki C, Hug M, Seifert B, Spahn DR, Beck-Schimmer B. Role of sevoflurane in organ protection during cardiac surgery in children: a randomized controlled trial. Interact Cardiovasc Thorac Surg. 2015 Feb;20(2):157-65.
- Walavalkar V, Evers E, Pujar S, Viralam K, Maiya S, Frerich S, John C, Rao S, Reddy C, Spronck B, Prinzen FW, Delhaas T, Vanagt WY. Preoperative Sildenafil administration in children undergoing cardiac surgery: a randomized controlled preconditioning study. Eur J Cardiothorac Surg. 2016 May;49(5):1403-10
- Yu D, Gong X, Zhang Y, Li Q, Zhang M. Tropisetron Preconditioning Decreases Myocardial Biomarkers in Patients Undergoing Heart Valve Replacement Surgery. Front Med (Lausanne). 2022 Mar 29;9:690272.

<u>No outcomes of interest</u>

- Lu EX, Chen SX, Yuan MD, Hu TH, Zhou HC, Luo WJ, Li GH, Xu LM. Preconditioning improves myocardial preservation in patients undergoing open heart operations. Ann Thorac Surg. 1997 Nov;64(5):1320-4.
- Hepponstall M, Ignjatovic V, Binos S, Attard C, Karlaftis V, d'Udekem Y, Monagle P, Konstantinov IE. Remote ischemic preconditioning (RIPC) modifies the plasma proteome in children undergoing repair of tetralogy of fallot: a randomized controlled trial. PLoS One. 2015 Mar 31;10(3):e0122778

- Struck R, Wittmann M, Recht T, Baumgarten G, Meybohm P, Müller A, Bagci S. Effect of remote ischemic preconditioning on the melatonin and antioxidative status: a pilot study in patients undergoing cardiac surgery. J Cardiovasc Surg (Torino). 2017 Dec;58(6):909-915
- Deja MA, Wiaderkiewicz R, Czekaj P, Czech E, Malinowski M, Machej L, Węglarzy A, Kowalówka A, Piekarska M, Szurlej B, Latusek T. Remote Ischaemic PrEconditioning of Human Myocardium (RIPE): study protocol for a double-blinded randomised controlled trial. Kardiol Pol. 2018;76(1):136-143
- Gaynor JW, Nicolson SC, Spray DM, Burnham NB, Chittams JL, Sammarco T, Walsh KW, Spray TL, Licht DJ. Remote Ischemic Preconditioning Does Not Prevent White Matter Injury in Neonates. Ann Thorac Surg. 2018 Jul;106(1):151-155
- Deja MA, Piekarska M, Malinowski M, Wiaderkiewicz R, Czekaj P, Machej L, Węglarzy A, Kowalówka A, Kołodziej T, Czech E, Plewka D, Mizia M, Latusek T, Szurlej B. Can human myocardium be remotely preconditioned? The results of a randomized controlled trial. Eur J Cardiothorac Surg. 2019 Jun 1;55(6):1086-1094.
- Drury NE, Bi R, Woolley RL, Stickley J, Morris KP, Montgomerie J, van Doorn C, Dunn WB, Madhani M, Ives NJ, Kirchhof P, Jones TJ. Bilateral Remote Ischaemic Conditioning in Children (BRICC) trial: protocol for a two-centre, double-blind, randomised controlled trial in young children undergoing cardiac surgery. BMJ Open. 2020 Oct 7;10(10):e042176.
- Drury NE, Menzies JC, Taylor CJ, Jones TJ, Lavis AC. Understanding parents' decision-making on participation in clinical trials in children's heart surgery: a qualitative study. BMJ Open. 2021 Feb 23;11(2):e044896

Review articles

- Scarci M, Fallouh HB, Young CP, Chambers DJ. Does intermittent cross-clamp fibrillation provide equivalent myocardial protection compared to cardioplegia in patients undergoing bypass graft revascularisation? Interact Cardiovasc Thorac Surg 2009 Nov;9(5):872-8
- Pilcher JM, Young P, Weatherall M, Rahman I, Bonser RS, Beasley RW. A systematic review and meta-analysis of the cardioprotective effects of remote ischaemic preconditioning in open cardiac surgery. J R Soc Med. 2012 Oct;105(10):436-45.
- Yang Y, Lang XB, Zhang P, Lv R, Wang YF, Chen JH. Remote ischemic preconditioning for prevention of acute kidney injury: a meta-analysis of randomized controlled trials. Am J Kidney Dis. 2014 Oct;64(4):574-83.
- Tie HT, Luo MZ, Li ZH, Wang Q, Wu QC. Remote ischemic preconditioning for pediatric patients undergoing congenital cardiac surgery: a meta-analysis. Int J Cardiol. 2014 Dec 15;177(2):551-3
- Tie HT, Luo MZ, Li ZH, Wang Q, Wu QC, Li Q, Zhang M. Remote Ischemic Preconditioning Fails to Benefit Pediatric Patients Undergoing Congenital Cardiac Surgery: A Meta-Analysis of Randomized Controlled Trials. Medicine (Baltimore). 2015 Oct;94(43):e1895
- Zhou C, Jeon Y, Meybohm P, Zarbock A, Young PJ, Li L, Hausenloy DJ. Renoprotection by remote ischemic conditioning during elective coronary revascularization: A systematic review and meta-analysis of randomized controlled trials. Int J Cardiol. 2016 Nov 1;222:295-302
- Tan W, Zhang C, Liu J, Li X, Chen Y, Miao Q. Remote Ischemic Preconditioning has a Cardioprotective Effect in Children in the Early Postoperative Phase: A Meta-Analysis of Randomized Controlled Trials. Pediatr Cardiol. 2018 Mar;39(3):617-626.

- Liu Z, Zhao Y, Lei M, Zhao G, Li D, Sun R, Liu X. Remote Ischemic Preconditioning to Prevent Acute Kidney Injury After Cardiac Surgery: A Meta-Analysis of Randomized Controlled Trials. Front Cardiovasc Med. 2021 Mar 18;8:601470
- Van den Eynde J, Cloet N, Van Lerberghe R, Sá MPBO, Vlasselaers D, Toelen J, Verbakel JY, Budts W, Gewillig M, Kutty S, Pottel H, Mekahli D. Strategies to Prevent Acute Kidney Injury after Pediatric Cardiac Surgery: A Network Meta-Analysis. Clin J Am Soc Nephrol. 2021 Oct;16(10):1480-1490
